# Survival after *Pneumocystis jirovecii* pneumonia requiring ventilation: A case report

**DOI:** 10.4102/sajhivmed.v17i1.474

**Published:** 2016-10-31

**Authors:** Gladness Nethathe, Nirav Patel

**Affiliations:** 1 Intensive Care Unit, Chris Hani Baragwanath Academic Hospital, South Africa; 2 Department of Pediatric Surgery, University of the Witwatersrand, South Africa

## Abstract

*Pneumocystis* pneumonia (PCP) in patients with the human immunodeficiency virus (HIV) is associated with a high mortality rate, which increases substantially with the need for mechanical ventilation. Local experience of patients with PCP admitted to the intensive care unit has revealed mortality rates close to 100%. We present a case of a 39-year-old HIV-infected man diagnosed with PCP who was successfully weaned from mechanical ventilation after presenting with respiratory distress and severe hypoxaemia. A short review of the literature will also be presented.

## Case

A 39-year-old man presented to the emergency department complaining of a 3-day history of shortness of breath and dry cough. He had no known significant co-morbidities and did not report any further associated symptoms. He reported that he was treated with antibiotics for a lower respiratory tract infection (LRTI) by a private physician in the previous 3 days with no improvement in symptoms. He was unsure of his HIV status and was not on antiretroviral therapy (ART).

His admission vitals were a blood pressure of 123/70 (82) mmHg, heart rate of 116 beats per minute, respiratory rate of 30 breaths per minute and a temperature of 36.9 °C. On examination, he had no significant lymphadenopathy and was not found to have mucosal or skin lesions associated with advanced HIV. No other obvious stigmata of advanced HIV were noted. He was in severe respiratory distress with scattered bilateral predominantly basal crackles on auscultation. Wheezing was not present on auscultation. He had an oxygen saturation of 62% on a partial rebreather mask with an inspired oxygen fraction (FiO_2_) of 80%.

Laboratory investigations revealed a white cell count (WCC) of 14.5 × 10^9^ cells/L, haemoglobin (Hb) of 11.6 g/L, platelets of 298 × 10^9^ cells/L, urea of 6.3 mmol/L, creatinine of 108 μmol/L, C-reactive protein (CRP) of 102 mg/L and beta-d-glucan (BDG) of > 500 pg/mL. His admission blood gas showed a mixed respiratory and metabolic acidosis with type II respiratory failure and a markedly increased alveolar-arterial gradient (pH = 7.36, pCO_2_ = 34.7, pO_2_ = 64.8, HCO_3_ = 19.2, BE = -4.9, SpO_2_ = 96.9, Hb = 13.4, Lac = 4.4). His chest X-ray (CXR) showed diffuse bilateral alveolar infiltrates and granular opacities. A transthoracic echocardiogram performed at the time of admission showed normal left ventricular function, no evidence of pulmonary embolus and normal heart valves. Based on clinical suspicion, hypoxaemic respiratory failure with typical chest radiograph changes, the patient’s normal echocardiogram, elevated BDG, and the relative absence of clinical signs usually associated with a multilobar pneumonia, the diagnosis of *Pneumocystis* pneumonia (PCP) was considered.

On the basis of his clinical condition and investigations, the patient was intubated, ventilated and admitted to the intensive care unit (ICU) for respiratory support. He was empirically started on treatment for partially treated community acquired pneumonia (CAP) (piperacillin and tazobactam and a macrolide) as well as PCP, on the basis of clinical case definition as suggested by the World Health Organisation (WHO), with high-dose intravenous trimethoprim-sulfamethoxazole (TMP-SMX) and high-dose intravenous corticosteroids (hydrocortisone). He consented to HIV testing on admission and was found to be HIV-infected with a CD4 count of 7 cells/μL. Additional sputum and blood investigations for *Mycobacterium tuberculosis* (TB) including TB culture (sputum and blood) and Auramine O stain, real-time polymerase chain reaction (PCR) (TB GeneXpert), sputum gram stain and bacterial culture and direct fluorescence antigen test for PCP were negative. Standard aerobic and anaerobic blood cultures yielded no pathogenic organisms.

The patient was intubated and ventilated for 3 days and spent a week in our unit. During this period, we adopted a lung protective ventilation strategy, permissive hypoxaemia as well as meticulous fluid management. Through his week-long admission, he had a cumulative fluid balance of -332 mL. Initial pre-admission ventilator settings of FiO_2_ = 1, PEEP = 14 and ΔP = 8 were successfully weaned to FiO_2_ = 0.6, PEEP = 10 and ΔP = 10 on day one of admission. We accepted oxygen saturations in the region of 70%–80% and PaO_2_ measurements in the region of 50 mmHg – 60 mmHg whilst maintaining tidal volumes of 4 mL/kg – 6 mL/kg. On day four of admission, despite oxygen saturations in the region of 80%, the patient was successfully extubated and placed on non-invasive ventilation (NIV). He required NIV (positive end expiratory pressure of 6 cm H_2_O and pressure support of 6 cm H_2_O) for a total of 3 days before being weaned onto oxygen supplementation with an FiO_2_ of 0.4. He maintained oxygen saturations in the region of 85%–90%. He was discharged to the ward on nasal cannula after a 7-day ICU stay.

## Discussion

### Literature search

A PubMed search was conducted to identify English language studies that have evaluated the ICU management of HIV-infected patients with PCP. Search terms used were ‘pneumocystis pneumonia and intensive care management and PCP and ventilatory management and HIV’. We additionally searched in the reference lists of relevant articles to identify further relevant literature.

PCP, once considered rare in Africa, is one of the most common opportunistic infections found in patients infected with HIV in developing countries.^1,2,3,4^ The prevalence of the disease in sub-Saharan African adults varies widely in clinical studies^3^ with more recent reports suggesting a high rate of clinical disease in African children.^5,6^ Hospital survival in HIV-infected adult patients with PCP ranges from 10% to 90%.^7,8^ The organism is classified as an opportunistic fungal pathogen of the genus *Pneumocystis* sp. It is an atypical fungus as it has a cell wall that contains cholesterol instead of ergosterol and does not grow in fungal culture.^9^ It exists in three forms, namely pre-cystic, cystic and trophic.^10^ Of the four species of *Pneumocystis*, *Pneumocystis jirovecii* is thought to be pathogenic only in humans, although *P. jirovecii* cannot be cultured in vitro.^11^ The acronym PCP is still used to refer to the clinical manifestation of the disease (PCP). Prior to the advent of HIV and/or AIDS PCP was usually only seen in immunosuppressed adults with malignancies or on corticosteroid therapy. More recently, the incidence of PCP has declined in developed countries as a result of widespread use of PCP prophylaxis and ART.^12,13^ In HIV-infected patients, the risk factors associated with *P. jirovecii* infection are CD4+ cell depletion as evidenced by CD4+ T-lymphocyte cell count < 200 cells/μL (200 × 10^6^ per L), previous *P. jirovecii* infection and other AIDS-defining illness.^14^

### Pathogenesis and clinical presentation

PCP typically presents as a non-productive cough, shortness of breath, fever and hypoxaemic respiratory failure with radiographic changes of alveolar infiltrates. This said, PCP may also present as a lobar pneumonia on chest radiography with few features of systemic inflammation. After inhalation, the organism attaches to type one alveolar cells resulting in injury to the alveolar epithelium.^15^ This attachment and the release of degradative enzymes from the pathogen is thought to play a role in initial alveolar damage.^16^ Initial damage to the epithelium results in an increase in alveolar capillary membrane permeability, which is in turn associated with an influx of inflammatory mediators such as neutrophils. Neutrophil-mediated mechanisms, such as the production of reactive oxygen species (ROS) (superoxide and hydrogen peroxide) and non-oxidative mechanisms, have been implicated in the pathogenesis of lung injury in PCP. Ultimately, PCP infection results in the production of an eosinophilic infiltrate, which fills the alveoli leading to impairment in oxygenation and resultant hypoxaemia, interstitial thickening and eventual fibrosis.^17,18^

Increasing experimental and clinical evidence supports the idea that lung damage occurring during PCP is the result of the type and extent of the host-mediated inflammatory response to infection rather than direct damage by the organism. Of particular note is the controversy surrounding the role of neutrophils and ROS in the inflammation and subsequent fibrosis observed in patients with PCP. Observations from human studies are that the severity of the disease correlates with the number of neutrophils in samples obtained through bronchoalveolar lavage (BAL) in both HIV-infected and non-HIV-infected patients with PCP.^19,20^ However, there is evidence to suggest that although neutrophils as well as their ROS are correlative markers of lung damage during *Pneumocystis* infection, they do not necessarily contribute to tissue damage.^21^ The clinical manifestation of the disease differs in immunosuppression from HIV compared to that from patients with other immunosuppressive conditions such as haematological malignancies.^22^ PCP in HIV-infected patients occurs at a time of profound immunosuppression from CD4+ T-cell depletion, whereas in patients receiving chemotherapy PCP typically occurs during the maintenance phase.^23^ The ability of patients without HIV-related illness or disease to mount an immune response may be associated with an exacerbated inflammatory response.^24^

### Diagnosis

The gold standard for diagnosing PCP is bronchoscopy with BAL and microscopic visualisation of the organism’s cystic or trophic forms using immunohistochemical stains.^25^ *P. jirovecii* cannot be cultured. PCP PCR assays are available and can be used in combination with BAL as well as on oral sputum or oral wash.^26,27,28,29^ Although *P. jirovecii* PCR sensitivity is reportedly high, it is non-specific as *P. jirovecii* colonisation or subclinical carriage is common.^11^

The use of biomarkers such as the BDG assay, a component of the cell wall of a number of medically important fungi, has also gained recent interest.^25^ In a study by Desmet et al.,^30^ BDG reactivity tested in serum samples from 28 patients with *Pneumocystis jirovecii* pneumonia (PCP) and 28 controls showed a sensitivity and specificity of BDG detection of 100% and 96.4%, respectively, using a cut-off value of 100 pg/mL. A limitation of this assay is that other fungal diseases may result in significantly raised levels.^30^ A negative serum BDG in HIV patients has been suggested as sufficient to rule out PCP.^31^ Other differentials that should be considered and excluded in this setting include mycobacterial infection (tuberculous and non-tuberculous), viral infections, toxoplasmosis and Kaposi’s sarcoma.

### Therapeutic options

Treatment for PCP should be started empirically if there is a high clinical suspicion. Confirmation of the diagnosis may be limited by factors such as availability of diagnostic tests. In such cases, the response to therapy may be used to guide management. Trimethoprim and sulfamethoxazole (TMP-SMX) is the treatment of choice for PCP as well as adjunctive corticosteroids for patients who present with hypoxaemia (PaO_2_ < 70 mmHg).^32^ The breakdown and clearance of the microbe may result in a severe inflammatory response in the lungs. Corticosteroid therapy has been shown to blunt this response and improve oxygenation.^33^ The widespread use of TMP-SMX prophylaxis allows for the possibility of resistance to sulphur drugs owing to mutations of the *Pneumocystis* dihydropteroate synthase gene. These mutations have been identified in up to 56% of *P. jirovecii* strains identified in South Africa.^14^ The clinical significance of this is unclear, and it is an area of debate whether this should alter pharmacological therapy.^34,35^ Clindamycin plus primaquine, atovaquone, pentamidine and trimetrexate are other effective options that may also be used.^36,37^ *P. jirovecii* is highly resistant to standard antifungal therapy such as azoles and amphotericin B because of the lack of ergosterol biosynthesis.^37^ Echinocandins, however, have activity against the cyst forms of the fungus as they target glucan synthesis in these fungal forms. Glucan presence is low in the trophic forms of the fungus.^38^ The trophic form is the predominant form during an infection. The use of ART is associated with decreased mortality in HIV-infected patients with PCP.^39,40^ Our patient was not on ART at the time of presentation.

## Strategies to improve hypoxaemia

Changes in the ventilatory and non-ventilatory management of patients with acute respiratory distress syndrome (ARDS) have improved survival in the last few decades. The use of lower tidal volumes and higher levels of positive end expiratory pressure in conjunction with other ventilatory (such as permissive hypercapnoea) and non-ventilatory strategies (such as fluid restriction) saw survival from ARDS improve from approximately 40% in 1991 to 60% in 1993 in developed regions.^41^ Patients with PCP-associated ARDS who require ventilation should be ventilated according to the ARDS Network guidelines using low tidal volumes and plateau pressures. The use of high flow nasal oxygen is emerging with new clinical applications and may provide an alternate method of oxygenation to NIV in this group of patients.^42^ Its use specifically in the management of hypoxaemia from PCP has not been adequately studied and was also not an available option in our unit at the time.

In patients with PCP, the prognosis is poor if ventilation is required, with a mortality rate that ranges from 40% to 100%.^40,41^ Early extubation may minimise superinfection, ventilator-associated pneumonia and other deleterious effects of intubation. The employment of permissive hypoxaemia may be prudent to minimise oxygen toxicity.

Lower mortality rates of patients with PCP admitted to the ICU with respiratory failure are noted in patients from developed regions though this may be due to differing admission criteria.^11,39,40^ The use of non-invasive mechanical ventilation as an alternative to endotracheal mechanical ventilation has been suggested as another possible explanation for the low 30-day mortality (33%) reported in some centres.^2^ In South Africa, PCP requiring ventilation is a suggested exclusion criterion for interventions such as extra-corporeal membrane oxygenation.^43^ The morbidity and mortality associated with PCP also appear to be related to the underlying cause of immunosuppression with patients with HIV-related illness or disease seeming to have much better outcomes than other groups of immunosuppressed patients such as those with malignancies or on corticosteroids.^24,44,45^ The prognosis of PCP has also been correlated with markers of inflammation supporting the idea that immune-mediated inflammation may be contributory to the pathogenesis of PCP.^39^

Sub-Saharan African literature on outcomes of HIV-associated respiratory failure from PCP requiring ventilation is limited. Interventions that can modify the disease process and improve outcomes of HIV-infected patients with PCP who require mechanical ventilation still need to be identified and further investigated. Management strategies such as permissive hypoxaemia, avoiding endotracheal intubation where possible, lung protective ventilation and meticulous fluid management seem to be appropriate in the management of hypoxaemia in this group of patients. Other strategies such as the use of high flow nasal oxygen in this group of patients require further exploration.
